# The second universal definition of heart failure (2026). What’s new?

**DOI:** 10.1093/eschf/xvag240

**Published:** 2026-09-16

**Authors:** Jan Biegus, Piotr Ponikowski

**Affiliations:** Department of Cardiology, Clinical Department of Intensive Cardiac Care, Faculty of Medicine, Wroclaw Medical University, Institute of Heart Diseases, Borowska 213, Wroclaw 50-556, Poland; Department of Cardiology, Jan Mikulicz Radecki University Hospital in Wrocław, Borowska 213, Wroclaw 50-556, Poland; Department of Cardiology, Clinical Department of Intensive Cardiac Care, Faculty of Medicine, Wroclaw Medical University, Institute of Heart Diseases, Borowska 213, Wroclaw 50-556, Poland; Department of Cardiology, Jan Mikulicz Radecki University Hospital in Wrocław, Borowska 213, Wroclaw 50-556, Poland

**Keywords:** Heart failure, Universal definition, Worsening heart failure, Decompensated heart failure

## Introduction

The first universal definition of heart failure (HF) published in 2021 was a document that addressed the fragmentation of HF nomenclature across guidelines, textbooks, and trial protocols by proposing a coherent understanding of HF as a syndrome of symptoms and/or signs caused by a structural and/or functional cardiac abnormality and corroborated by elevated natriuretic peptides or objective evidence of congestion.^1^ Five years on, the case for revising the initial document rests on a globally rising prevalence of the disease, driven largely by HFpEF and population aging; advances in imaging and genetics that multiply the number of recognizable phenotypes; accumulating evidence of benefit outside narrow LVEF bands; and well-documented inequities in access to diagnosis and treatment.^2^

## The core definition

The major syndrome definition remains rather unchanged. Heart failure is a clinical syndrome of diverse causes, characterized by (typical, but not specific) symptoms, signs, and laboratory or imaging abnormalities suggesting congestion or altered cardiac output, attributable to a structural or functional cardiac abnormality. As stated in the document: ‘… HF is defined by the accumulation of suggestive features in a comprehensive assessment rather than by a single diagnostic test.’ Elevated natriuretic peptide levels increase the likelihood of the HF diagnosis.^2^

The change from the previous document is subtle but important. The 2021 text framed objective corroboration as a requirement and operationalized the structural abnormality through explicit thresholds (EF <50%, chamber enlargement, E/E′ > 15, moderate/severe hypertrophy, valvular lesion). The 2026 version no longer repeats the numerical operationalization and instead emphasizes that HF is defined by the accumulation of suggestive features in a comprehensive assessment, rather than by any single test. The argument is HFpEF: a substantial proportion of patients may have normal natriuretic peptides despite unequivocal invasive evidence of elevated filling pressures at rest or on exercise. Practically, normal natriuretic peptide concentration should not be considered sufficient grounds to close the workup in a breathless, obese patient with a typical haemodynamic profile.

The document also reaffirms the permanence of the diagnosis: once symptomatic HF has been established, the diagnosis is retained for life, irrespective of subsequent clinical or functional improvement.

## The end of fixed LVEF cut-offs?

The first definition introduced four possible HF presentations based on left ventricular ejection fraction (LVEF) cut-offs: HFrEF (≤40%), HFmrEF (41%–49%), HFpEF (≥50%), and HFimpEF. The second definition calls those cut-offs arbitrary, citing the variability of echocardiographic measurement, the difficulty of comparing LVEF across imaging modalities, and the unresolved question of whether cut-offs should account for sex, age, and ethnicity.

The reference argument is simple and forceful. The lower limit of normal LVEF is ∼53% in women and 52% in men, slightly higher in individuals of Asian origin; an LVEF below 50% is therefore unlikely to reflect normal function. If so, a separate category for the 41%–49% band becomes hard to defend (in the context of diagnosis and definition)—particularly given growing evidence that patient with mildly reduced LVEF benefit from standard HFrEF therapies. The classification now comprises three clinically actionable groups but without providing fixed cut-offs between HFrEF and HFpEF (*Figure 1*):

- HFrEF—heart failure with reduced ejection fraction;
- HFpEF—heart failure with preserved ejection fraction;
- HFimpEF—previously reduced LVEF that has now increased or normalized.

**Figure 1:**
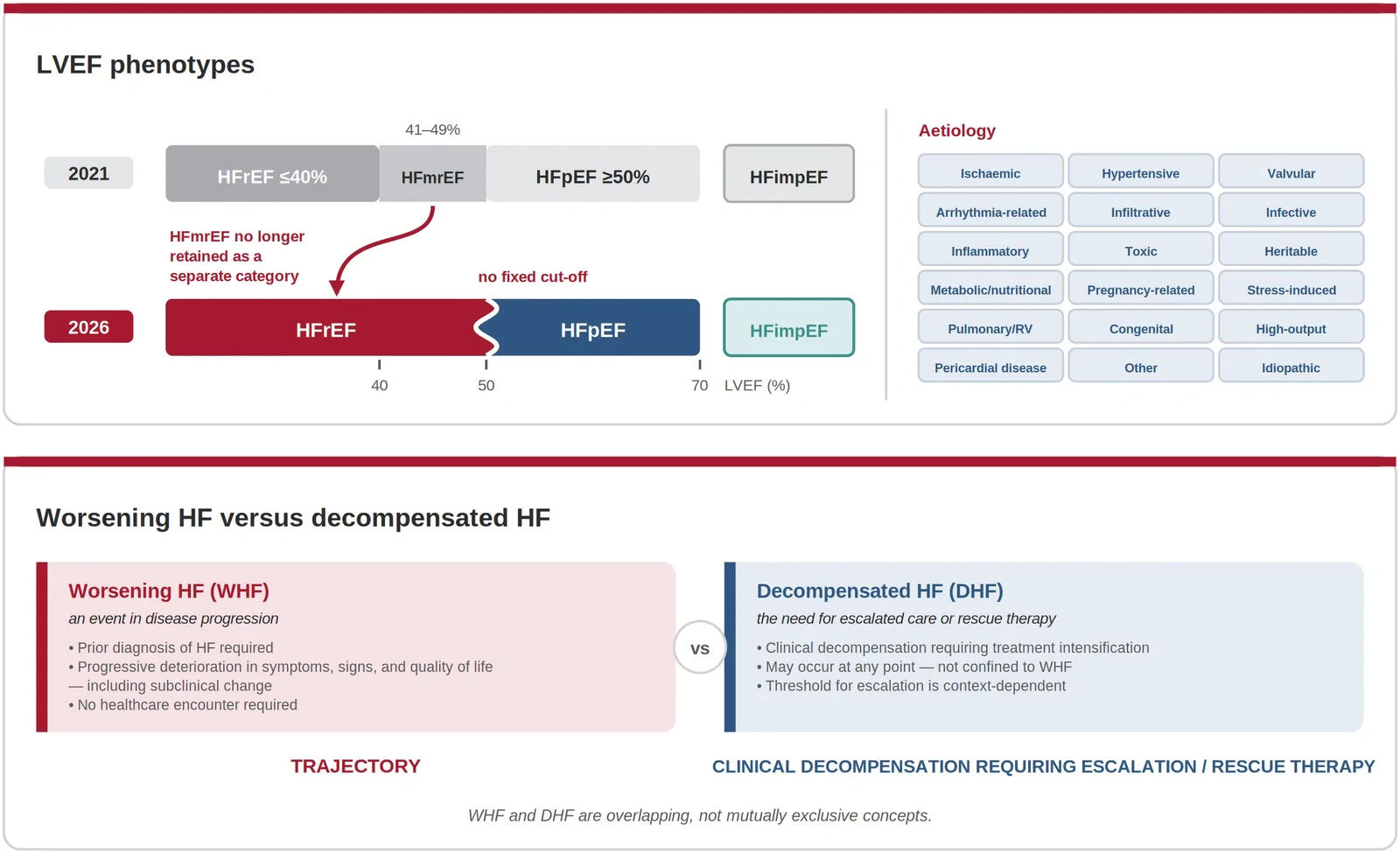
The summary of key new concepts in the Second Universal Definition of Heart Failure

The definition of HFimpEF remains quantitative (antecedent HFrEF, then a ≥10%-point rise to >40%), but the document stresses that an exclusively LVEF-based definition insufficiently reflects the broader phenotype—persistent structural abnormalities, biomarker elevation, or residual symptoms.

## Aetiology is promoted to classification

The 2021 document emphasized the importance of identifying the aetiology of HF but did not propose a universal classification of its causes. Closing that gap is the second definition’s methodological contribution. Its starting point is a critique of the entrenched ischaemic/non-ischaemic dichotomy, which omits the many causes of dilated and hypertrophic cardiomyopathy and fails to inform treatment. Cardiac amyloidosis remains the paradigm case: a diagnosis stopping at ‘HFpEF’ denies the patient targeted, potentially life-prolonging therapy.

The proposed classification spans eighteen pathogenic groups: ischaemic, hypertensive, valvular and arrhythmia-related; infiltrative, infective, inflammatory, toxic and heritable; metabolic and nutritional, pregnancy-related, stress-induced, pulmonary/right-sided, congenital, pericardial disease and high-output mediated; plus ‘other’ and ‘idiopathic’.

## Stages of heart failure

The A–D framework is reaffirmed without any major changes, retaining the renaming of stage B to pre-HF. The document also emphasizes a risk gradient between stages A and B, justifying more intensive proactive strategies at stage B, including renin–angiotensin–aldosterone system inhibitors and beta-blockers in asymptomatic left ventricular dysfunction.

## Worsening vs decompensated HF

This is the change with the greatest ordering potential for clinical research. Worsening HF (WHF) is a clinically detectable event in disease progression: progressive deterioration in symptoms, signs and quality of life in someone with a prior diagnosis of HF. It therefore excludes new-onset HF and deterioration attributed primarily to unrelated precipitants, like acute coronary syndromes, infection, poor adherence. Manifestations span dyspnoea and oedema but also malignant arrhythmias, reduced exercise capacity and subclinical change: rising pulmonary pressures, impaired ventricular function on imaging, and rising natriuretic peptides and troponins. Critically for ambulatory trial design, a healthcare encounter is not a prerequisite.

Decompensated HF (DHF), when compared with WHF, is defined by the need for escalated care or rescue therapy, may occur at any point and, in contrast to WHF, can be diagnosed in patients with and without previously diagnosed HF. The 2026 document prefers the DHF nomenclature over acute heart failure (AHF), which was used in recent ESC guidelines.

Its defining feature is treatment modification in response to clinical decompensation: increased or combination diuretic therapy, implementation of missing guideline-directed therapy, and in severe cases resynchronization, valvular intervention, mechanical support or transplantation. The document notes that the threshold for care escalation (rescue therapy) varies with regional practice and resources, making DHF context dependent.

## Prevention and early detection

The preventive catalogue expands: alongside SGLT2 inhibitors, GLP-1 receptor agonists and finerenone in CKD with diabetes are named, and obesity acquires standing as a pharmacotherapy target shown to reduce new-onset HF. Screening still rests on natriuretic peptides plus risk factor assessment, with troponin and urine ACR complementary in higher-risk groups.

## Conclusions

The Second Universal Definition moves the centre of gravity from a taxonomy built on numbers towards a classification that is clinically actionable, aetiologically grounded and attentive to time. Three LVEF groups instead of four, the first universal classification of causes, the separation of worsening from DHF. The authors describe it as a living framework. That is the right posture: in a disease whose nature is dynamic, a static definition would be a contradiction in terms.

## Declarations

### Data Availability

This article did not involve the generation or analysis of any original data. Therefore, no data is available for sharing.
